# Correction to “The Myokine Irisin Represents an Indirect Pathway Linking Exercise to Hippocampal Subfields Relevant to Alzheimer's Disease and Neurogenesis”

**DOI:** 10.1111/acel.70591

**Published:** 2026-06-16

Pace, T. , J. M. Levenstein , B. L. Quigley , R. Houston , A. P. Bouças , and S. C. Andrews . (2026). The Myokine Irisin Represents an Indirect Pathway Linking Exercise to Hippocampal Subfields Relevant to Alzheimer's Disease and Neurogenesis. Aging Cell 25, no. 5: e70497. 10.1111/acel.70497.42033046 PMC13109656

In the published article, the commercial ELISA kit used to measure circulating irisin was reported incorrectly. The kit used in the present study was Phoenix Pharmaceuticals catalogue number EK‐067‐29, not EK‐067‐52. The Methods section reported the quantifiable range associated with EK‐067‐52, and the Discussion section named EK‐067‐52 as the kit used in the validation passages. The corrections below identify each affected passage and provide the corrected text. All 74 samples were assayed using a single lot of EK‐067‐29 under identical conditions, and all irisin values were natural log‐transformed and z‐scored prior to analysis. Every measurement, analysis, and reported result was derived from the EK‐067‐29 assay throughout, so correcting the catalogue label in the text does not alter any raw value, statistical model, or finding. The results, statistical analyses, figures, and conclusions of the article remain as published. The minor changes are shown below:

Section 2.2.1. The text: “Serum irisin levels were measured in duplicate and averaged using the Irisin ELISA kit (Human, Rat, Mouse, Canine; Phoenix Pharmaceuticals, California, USA) following the manufacturer's instructions. All study samples yielded detectable levels of irisin within the kit's quantifiable range (0.066–1024 ng/mL).” was incorrect.

This should have read: “Serum irisin levels were measured in duplicate and averaged using the Irisin ELISA kit (*catalogue number EK‐067‐29*; Human, Rat, Mouse, Canine; Phoenix Pharmaceuticals, California, USA) following the manufacturer's instructions. All study samples yielded detectable levels of irisin within the kit's quantifiable range (*0.1–1000* ng/mL).”

Section 4.5. The text: “These lot‐to‐lot concerns were raised with respect to older Phoenix immunoassays including earlier versions of the EK‐067‐52; subsequent independent evaluation explicitly concluded that EK‐067‐52 was the most thoroughly validated commercial irisin ELISA kit available (Perakakis et al. 2017).” was incorrect.

This should have read: “These lot‐to‐lot concerns were raised with respect to older Phoenix immunoassays including earlier versions of the *EK‐067‐29*; subsequent independent evaluation explicitly concluded that *EK‐067‐29* was the *leading* commercial irisin ELISA kit available *at the time of review* (Perakakis et al. 2017).”

Section 4.5. The text: “Our exercise‐irisin association (*β* = 0.365, *p* = 0.003) is consistent with meta‐analytic evidence that acute exercise produces reliable increases in circulating irisin (Fox et al. 2018), including independent replications using the same assay kit (Daskalopoulou et al. 2014; Qiu et al. 2020).” was incorrect.

This should have read: “Our exercise‐irisin association (*β* = 0.365, *p* = 0.003) is consistent with meta‐analytic evidence that acute exercise produces reliable increases in circulating irisin (Fox et al. 2018), including *studies using a related Phoenix immunoassay* (Daskalopoulou et al. 2014; Qiu et al. 2020), *and including studies using EK‐067‐29 specifically*.”

Section 4.5. The text: “Beyond these internal features of our data, the kit itself has independent validation support. A head‐to‐head comparison of three commercial irisin assays judged the kit used in the present study (EK‐067‐52) as best suited for clinical sample analysis, based on its wider optical density spectrum and the consistent linearity of human irisin values within the standard curve (Park et al. 2013). A subsequent independent review of commercially available irisin ELISAs reached the same conclusion, identifying EK‐067‐52 as the most validated commercial irisin ELISA kit available (Perakakis et al. 2017). A related Phoenix antibody used is the EK‐067‐29 kit, which has additionally been shown to detect a protein sequence matching irisin identified by MALDI‐TOF mass spectrometry (Perakakis et al. 2017; Zhang et al. 2014), suggesting that Phoenix antibodies bind genuine irisin even if absolute values are inflated by additional non‐specific signal.” was incorrect.

This should have read: “Beyond these internal features of our data, the kit itself has independent validation support. A head‐to‐head comparison of three commercial irisin assays judged *a closely related Phoenix kit* (EK‐067‐52) as best suited for clinical sample analysis, based on its wider optical density spectrum and the consistent linearity of human irisin values within the standard curve (Park et al. 2013). A subsequent independent review of commercially available irisin ELISAs reached the same conclusion, identifying EK‐067‐52 as the most validated commercial irisin ELISA kit available, *and identified EK‐067‐29, the kit used in the present study, as the leading commercial kit available at the time of review* (Perakakis et al. 2017). *The antibody used in* EK‐067‐29 has additionally been shown to detect a protein sequence matching irisin identified by MALDI‐TOF mass spectrometry (Perakakis et al. 2017; Zhang et al. 2014), suggesting that Phoenix antibodies bind genuine irisin even if absolute values are inflated by additional non‐specific signal.”

We apologize for this error.
